# Photosynthetic and Chlorophyll Fluorescence Characteristics of *Isodon rubescens* (Hemsley) H. Hara

**DOI:** 10.1038/s41598-020-67192-2

**Published:** 2020-06-22

**Authors:** Jian Zaiyou, Zhou Xiuren, Tian Jing

**Affiliations:** Henan Institute of Science and Technology, Xinxiang, China 453003

**Keywords:** Ecophysiology, Plant physiology

## Abstract

The ecological and economic cultivation of *Isodon rubescens* is currently being carried out. The demand of *I. rubescens* for light intensity should be made clear to estimate whether the environmental conditions of an area are suitable for cultivating *I. Rubescens* and improve cultivation techniques. The photosynthetic and chlorophyll fluorescence characteristics of *I. rubescens* were determined with a Li-6400 photosynthesis system and PAM-2500 portable chlorophyll fluorescence apparatus. The results showed that there was no obvious midday depression of photosynthesis in *I. rubescens* leaves. The light compensation point and light saturation point of *I. rubescens* leaves were 21.83482 µmol·m^−2^·s^−1^ and 802.262 µmol·m^−2^·s^−1^, respectively. The CO_2_ compensation point and CO_2_ saturation point of *I. rubescens* leaves were 101.7199 µmol·mol^−1^ and 1674.514 µmol·mol^−1^, respectively. The maximal photochemical efficiency of photosystem II ((Fm-Fo)/Fm) in *I. rubescens* leaves reached 0.7. The electron transport rate of photosystem II in *I. rubescens* leaves reached 20 μmol electrons/(m^2^·s). *I. rubescens* can tolerate intense light above the light compensation point and utilize low light. *I. rubescens* leaves have a strong photoprotective capacity. *I. rubescens* can grow in both sunny and shady places. The most important factor affecting photosynthetic efficiency in *I. rubescens* leaves is the concentration of CO_2_ in air.

## Introduction

*Isodon rubescens* (Hemsley) H. Hara is a perennial subshrub belonging to a genus of the Lamiaceae family^1^. There are several bioactive chemical components in *I. rubescens*, such as oridonin and ponicidin. The dry aerial portions of *I. rubescens* are named rabdosiae rubescentis herba and are used in traditional Chinese medicine for the treatment of sore throats, inflammation and gastrointestinal problems^2,3^.

The ecological and economic cultivation of *I. rubescens* is currently being carried out. However, there are different environmental conditions in different places. Wild *I. rubescens* grows on mountains or hills. There are obvious differences between the environmental conditions of mountains and plains. The demand of *I. rubescens* for light intensity should be made clear. The photosynthetic and chlorophyll fluorescence characteristics of *I. rubescens* were studied in this research to define the most suitable environmental conditions for *I. rubescens* cultivation and improve cultivation techniques.

## Results

### Diurnal variation in *I. rubescens* leaf photosynthesis

**Figure 1:**
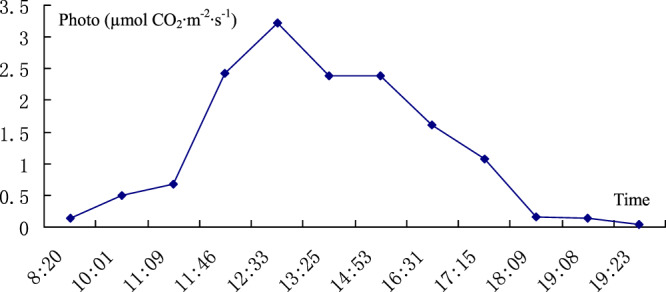
Diurnal variation in *I. rubescens* leaf photosynthesis.

The results of the diurnal variation in *I. rubescens* leaf photosynthesis are shown in Table 1. Based on the collected data, the curve of the diurnal variation in *I. rubescens* leaf photosynthesis is shown in Fig. 1.

**Table 1:** Diurnal variation in *I. rubescens* leaf photosynthesis (average).

Time	Photo (µmol CO_2_·m^−2^·s^−1^)	Transpiration (mmol H_2_O m^−2^s^−1^)	PARout (µmol·m^−2^·s^−1^)	Tleaf (°C)	CO_2_ (µmol·mol^−1^)
8:20	0.134554	0.078752	165.3032	27.48817	374.3593
10:01	0.489859	0.100056	690.494	31.51378	364.5589
11:09	0.677691	0.16249	923.3254	33.61967	384.1379
11:46	2.431713	2.894369	1211.345	34.83849	370.7948
12:33	3.21911	2.629756	1484.135	35.78894	361.1602
13:25	2.385099	2.6251	1697.34	36.47706	363.1944
14:53	2.390117	2.888978	1346.388	35.82655	370.8038
16:31	1.619721	2.060572	524.8741	33.17798	359.4734
17:15	1.067346	1.362125	280.3203	32.90131	359.0663
18:09	0.166573	0.043506	163.6786	31.8848	370.7527
19:08	0.146304	0.063026	145.7678	30.09128	366.364
19:23	0.038786	0.050609	86.26784	29.08384	366.6463

Note: PARout is the PAR out of the leaf chamber.

The diurnal variation in *I. rubescens* leaf photosynthesis indicates that there was no obvious midday depression of photosynthesis. There is still a high net photosynthetic rate in *I. rubescens* leaves at noon with high light intensity. Leaves of *I. rubescens* can utilize very faint light, e.g., with a 20 µmol·m^−2^·s^−1^ intensity. The leaves of *I. rubescens* can photosynthesize even in the faint light of evening.

### Light response curve of *I. rubescens* leaves

**Figure 2:**
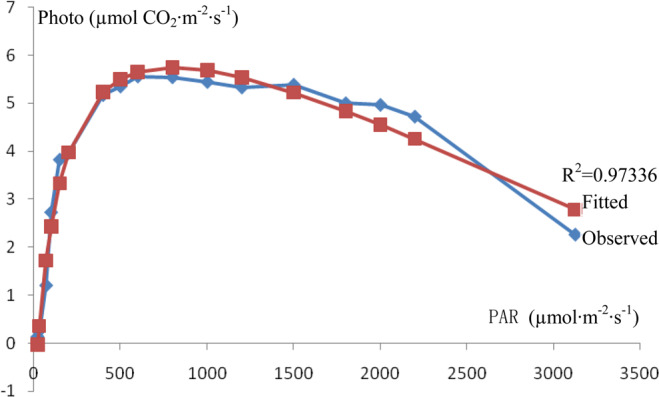
Light response curve of *I. rubescens* leaf photosynthesis.

The data from the light response curve of *I. rubescens* leaf photosynthesis are shown in Table 2. Based on the collected data, the curve of the light response of *I. rubescens* leaf photosynthesis is shown in Fig. 2.

**Table 2:** Light response curve of *I. rubescens* leaf photosynthesis (average).

PARin (µmol·m^−2^·s^−1^)	Photo (µmol·m^−2^·s^−1^)	Transpiration (mmol m^−2^s^−1^)	Tleaf (°C)	CO_2_ (µmol·mol^−1^)
3124.457	2.260209	4.466418	29.3982	395.2443
2198.982	4.721694	3.573953	30.04687	392.505
2000.081	4.965527	3.807614	30.61862	391.8968
1800.327	5.001151	3.645889	29.77489	392.8282
1500.057	5.38657	3.336598	30.33475	392.6403
1200.517	5.329608	2.987329	30.81813	391.6764
1001.321	5.443283	2.672035	30.69158	391.8007
799.9315	5.541983	2.354444	30.53654	391.9345
599.9705	5.559295	2.089824	30.37206	391.9419
499.3938	5.348708	1.890056	30.96318	392.7756
400.1194	5.171424	1.844144	30.15175	393.0815
200.3279	3.963617	1.612985	30.92859	394.4694
149.6206	3.822843	1.509174	30.68748	395.1866
99.69547	2.725072	1.457733	30.33666	396.0288
70.8973	1.200702	1.417435	30.03102	398.5548
30.33922	0.248815	1.142081	29.94907	399.1747
21.26013	0.1307	1.322936	29.69206	399.0078
E	0.050712			
M	0.00021			
N	0.005891			
LCP	21.83482 µmol·m^−2^·s^−1^			
E·LCP	1.107			
LSP	802.262 µmol·m^−2^·s^−1^			
PLSP	5.7471 µmol·m^−2^·s^−1^			
R^2^	0.97336			

Note: PARin is the PAR in the leaf chamber. PLSP is the net photosynthetic rate at the light saturation point.

The light response curve of *I. rubescens* leaf photosynthesis indicates that the net photosynthetic rate was obviously related to the light intensity when the light intensity was low. The net photosynthetic rate of *I. rubescens* leaves rapidly increased as the light intensity increased from 20–400 µmol·m^−2^·s^−1^. *I. rubescens* leaves were able to utilize intense light. With light intensities of 400–2200 µmol·m^−2^·s^−1^, the net photosynthetic rate of *I. rubescens* leaves was high. However, the net photosynthetic rate of *I. rubescens* leaves obviously decreased when the light intensity was above 2200 µmol·m^−2^·s^−1^.

The results of the light response curve fitted with the modified rectangular hyperbola model are shown in Table 2. The fitted light saturation point and the net photosynthetic rate at this point were very similar to the observed value.

### CO_2_ response curve of *I. rubescens* leaves

The CO_2_ response curve data of *I. rubescens* leaf photosynthesis are shown in Table 3.

**Table 3:** CO_2_ response curve of *I. rubescens* leaf photosynthesis (average).

CO_2_ (µmol·mol^−1^)	Photo (µmolCO_2_·m^-−2^·s^−1^)	Transpiration (mmolH_2_Om^−2^s^−1^)	Tleaf (°C)	PARin (µmol·m^−2^·s^−1^)
1981.063	13.28118	1.711614	30.67786	1200.049
1779.07	13.74822	2.076798	30.12322	1200.109
1477.98	13.9082	2.422849	30.99781	1199.903
1381.585	12.22414	2.429738	30.03088	1199.725
1181.821	11.9153	2.542288	30.79212	1198.881
983.2891	11.6798	2.593174	30.62088	1199.717
785.1578	10.43091	2.605263	30.6294	1199.769
588.2518	8.370093	2.598826	29.70778	1199.477
392.7176	5.688908	2.572764	29.82269	1199.592
361.7573	3.743928	2.235932	30.30235	1200.262
286.5714	2.378747	2.31753	30.69262	1200.178
256.2721	2.153212	2.340206	30.60159	1199.589
197.9524	1.113644	1.946278	30.54132	1201.117
148.4747	0.958305	2.032948	30.34006	1201.138
99.09576	0.396179	2.113611	30.11915	1201.403
69.56865	0.095351	2.193027	29.91523	1201.294
E	0.050712			
M	0.00021			
N	0.005891			
CCP	101.7199 µmol·mol^−1^			
E·CCP	1.107			
CSP	1674.514 µmol·mol^−1^			
PCSP	13.62882 µmol·m^−2^·s^−1^			
R^2^	0.98526			

Note: CCP is the CO_2_ compensation point. CSP is the CO_2_ saturation point. PCSP is the net photosynthetic rate at the CO_2_ saturation point. PARin is the PAR in the leaf chamber.

**Figure 3:**
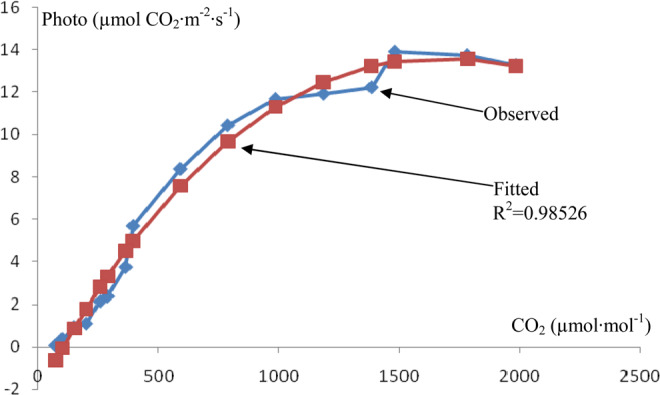
CO_2_ response curve of *I. rubescens* leaf photosynthesis.

Based on the collected data, the curve of the CO_2_ response of *I. rubescens* leaf photosynthesis is shown in Fig. 3.

The CO_2_ response curve of *I. rubescens* leaf photosynthesis indicates that the net photosynthetic rate was obviously related to the concentration of CO_2_ in the air when the CO_2_ concentration was below 1000 µmol·mol^−1^. However, the effect of the CO_2_ concentration on the net photosynthetic rate was not obvious when the concentration of CO_2_ was above 1000 µmol·mol^−1^.

The results of the CO_2_ response curve fitted with the modified rectangular hyperbola model are shown in Table 3. The fitted CO_2_ saturation point and the net photosynthetic rate at this point were very similar to the observed value.

### Chlorophyll fluorescence characteristics of *I. rubescens l*eaves

The results of the slow kinetics of chlorophyll fluorescence are shown in Table 4.

**Table 4:** Slow kinetics of chlorophyll fluorescence.

PAR (µmol·m^−2^·s^−1^)	(Fm-Fo)/Fm	Y(II)	Y(NPQ)	Y(NO)	qN	qP	ETR (μmol electrons·m^−2^·s^−1^
198	0.725319	0.25	0.468	0.282	0.755	0.528	20.8
198	0.75982	0.239	0.534	0.227	0.835	0.57	19.9
198	0.727212	0.274	0.453	0.273	0.731	0.527	22.8
Average	0.7374	0.2543	0.485	0.261	0.7737	0.5417	21.167

The slow kinetics of chlorophyll fluorescence of *I. rubescens* leaves indicates that the maximal photochemical efficiency of photosystem II ((Fm-Fo)/Fm) in *I. rubescens* leaves reached 0.7. The electron transport rate of photosystem II in *I. rubescens* leaves reached 20 μmol electrons/(m^2^·s). The fraction of energy dissipated as heat via the regulated photoprotective NPQ mechanism (Y(NPQ)) was much more than that passively dissipated in the form of heat and fluorescence (Y(NO)).

**Figure 4:**
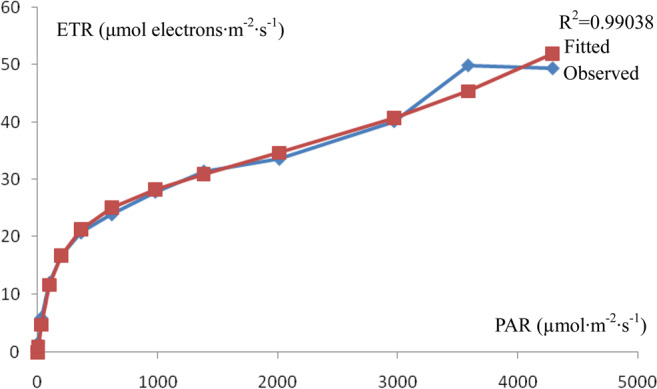
Rapid light curve of chlorophyll fluorescence in *I. rubescens* leaves.

The results of the rapid light curves of chlorophyll fluorescence in *I. rubescens* leaves are shown in Table 5. The rapid light curve of chlorophyll fluorescence in *I. rubescens* leaves is shown in Fig. 4.

**Table 5:** Rapid light curve of chlorophyll fluorescence in *I. rubescens* leaves (average).

PAR	Y(II)	Y(NPQ)	Y(NO)	NPQ	qN	qP	qL	ETR
0	0.7900	0.000	0.210	0.000	0.000	1.0000	1.0000	0.00
6	0.6763	0.018	0.306	0.059	0.067	0.8670	0.5890	1.70
31	0.4633	0.206	0.330	0.644	0.458	0.6663	0.3803	6.03
101	0.2837	0.421	0.295	1.456	0.683	0.4687	0.2593	12.03
198	0.2007	0.531	0.268	1.997	0.763	0.3597	0.2000	16.70
363	0.1363	0.601	0.263	2.299	0.795	0.2550	0.1377	20.77
619	0.0920	0.646	0.262	2.465	0.822	0.1877	0.1047	23.93
981	0.0677	0.669	0.263	2.546	0.828	0.1393	0.0767	27.87
1386	0.0540	0.684	0.262	2.617	0.833	0.1120	0.0613	31.43
2015	0.0397	0.699	0.261	2.682	0.837	0.0837	0.0457	33.60
2970	0.0320	0.708	0.260	2.734	0.842	0.0690	0.0380	40.17
3588	0.033	0.716	0.251	2.849	0.834	0.065	0.033	49.9
4292	0.027	0.725	0.248	2.922	0.838	0.054	0.027	49.4
a	−0.00000359906							
b	0.033474211							
c	5.362466019							
Fv’/Fm’ x ETR factor/2		0.33167 electrons/photons						
Alpha		0.08 electrons/photons						
ETRmax		52.333μmol electrons/(m^2^·s)						
Ik		705.3μmol electrons/(m^2^·s)						
Fv’/Fm’		0.78988						
R^2^		0.99038						

Note: Fv’/Fm’ x ETR factor/2 is the maximum quantum yield of PSII with a saturated pulse after dark adaptation. Alpha is the initial slope. ETRmax is the maximum electron transport rate. Ik is the minimum saturation of the light intensity.

The rapid light curve of chlorophyll fluorescence in *I. rubescens* leaves was automatically fitted with a PAM-2500 portable chlorophyll fluorescence apparatus according to the model of Eilers and Peeters [5]. The fitted results are shown in Table 5.

The rapid light curve of chlorophyll fluorescence in *I. rubescens* leaves indicates that the maximum quantum yield of PSII with a saturated pulse after dark adaptation (Fv’/Fm’ x ETR factor/2) was higher than the effective quantum yield of PSII (Y(II)). The initial slope (alpha) signifying the maximum photosynthetic efficiency was higher than the apparent quantum yield fitted in the light response curve of *I. rubescens* leaves.

## Discussion and Conclusion

The modified rectangular hyperbola model is suitable for fitting light response curves and CO_2_ response curves. We compared the fit of the light response curve and CO_2_ response curve of *Paeonia lactiflora* created with different models. It was found that the fit results based on the modified rectangular hyperbola model were more similar than the results from other models to the observed values^6^.

*I. rubescens* is a heliophyte plant, which can tolerate intense light. There are very few reports about photosynthesis of *I. Rubescens*. There was no obvious midday depression of photosynthesis in *I. rubescens* leaves in terms of this study. The midday photosynthetic depression occurred in most of plants. The factors such as intense light, high air temperature, low soil moisture, low air humidity and so on can cause midday photosynthetic depression^7–10^. There is no midday photosynthetic depression in some other plants, such as C_4_ plants (Characterized by the Hatch-Slack photosynthetic pathway), CAM plants (plants with crassulacean acid metabolism) and aquatic plant^11,12^. Some plants perform midday photosynthetic depression in a certain environment but express no midday photosynthetic depression in another environment. Their performances are affected by environment or some chemicals^13–16^. The environment of *I. rubescens* studied in this paper was consistent with that of yield *I. rubescens*. It was sunny day and the light intensity was highest in a year in the locality when the data were determined. *I. rubescens* performed no midday photosynthetic depression in the severe environment, which indicated that it would similarly perform in suitable environment. Therefore, *I. rubescens* can tolerate intense light.

There was no obvious difference between the net photosynthetic rate of light saturation point and that of light intensities of 2000 µmol·m^−2^·s^−1^ although the light saturation point of *I. rubescens* leaves was 802.262 µmol·m^−2^·s^−1^. Therefore, there was no obvious effect of intense light above the light saturation point on the photosynthesis of *I. rubescens* leaves. The net photosynthetic rate of the light intensities of 1484.135 µmol·m^−2^·s^−1^ was the highest in diurnal variation of photosynthesis because the temperature was suitable for it at that time. *I. rubescens* can also tolerate low light. Leaves of *I. rubescens* can utilize low light (i.e., at an intensity of 20 µmol·m^−2^·s^−1^). Therefore, *I. rubescens* can grow on shady slopes. The most important factor affecting the photosynthetic efficiency in *I. rubescens* leaves is the concentration of CO_2_ in the air. Photosynthesis in *I. rubescens* leaves was not obviously affected by high concentrations of CO_2_ alone.

The maximum electron transport rate (ETRmax) in *I. rubescens* leaves was far higher than the observed electron transport rate (ETR). The chlorophyll fluorescence characteristics of *I. rubescens* leaves showed that there was very large potential for photosynthesis in *I. rubescens* leaves. The fraction of energy dissipated as heat via the regulated photoprotective NPQ mechanism (Y(NPQ)) was much more than that passively dissipated in the form of heat and fluorescence (Y(NO)). The minimum saturation light intensity (Ik) was far less than the light saturation point (LSP). Therefore, *I. rubescens* leaves can tolerate intense light.

*I. rubescens* performs no midday photosynthetic depression and can tolerate intense light. It can utilize low light and possesses high value of Fv/ Fm (the maximal photochemical efficiency of photosystem II). This indicated that *I. rubescens* leaves have a strong photoprotective capacity. However, the growth and cultivation of *I. rubescens* are affected by many factors such as light, air temperature, rainfall, soil, and so on^17,18^. This study is aimed at the photosynthetic and chlorophyll fluorescence characteristics of *I. rubescens*. The suitable environment for the growth and cultivation of *I. rubescens* still needs to study.

## Materials and Methods

### Instruments

Li-6400 Photosynthesis system (LI-6400 Inc., Lincoln, NE, USA). PAM-2500 portable chlorophyll fluorescence apparatus (PAM-2500, Walz, Germany).

### Materials

Approximately 60 *I. rubescens* plants were dug up from Taihang Mountain and evenly planted in 12 flowerpots (30 cm in diameter and 35 cm in depth) in March 2018. Then, the plants were irrigated to ensure that they grew well.

### Determination of photosynthetic characteristics

The photosynthetic characteristics of mature leaves on the *I. rubescens* plants were determined on June 5–7 (sunny day, the light intensity is highest in a year), 2019. The concentration of CO_2_ in the air was approximately 370 µmol·mol^−1^ when the diurnal variation of photosynthesis was determined. The temperature of the leaf chamber was set at 30 °C, and the concentration of CO_2_ in the leaf chamber was set at 400 µmol·mol^−1^ when the light response curve was determined. The light intensity in the leaf chamber was set at 1200 µmol·m^−2^·s^−1^, and the temperature of the leaf chamber was set at 30 °C when the CO_2_ response curve was determined. These photosynthetic characteristics were determined with the Li-6400 Photosynthesis system. Each determination was repeated three times.

### Determination of chlorophyll fluorescence characteristics

The fluorescence characteristics of mature leaves on the *I. rubescens* plants were determined on June 7–8, 2019. The leaves were under dark adaptation for 30 min before the determination of the chlorophyll fluorescence characteristics. The slow kinetics of chlorophyll fluorescence were determined before determining the light curve of chlorophyll fluorescence. The tests were repeated three times.

### Data analysis

The light response curve and CO_2_ response curves were analysed with SPSS (Statistical Product and Service Solutions, International Business Machines Corporation, USA). The light response curve and CO_2_ response curve were all fitted with a modified rectangular hyperbola model^4^.

Modified rectangular hyperbola model:

$${\rm{Photo}}={\rm{E}}\cdot (1-{\rm{M}}\cdot {\rm{PAR}})\cdot ({\rm{PAR}}-{\rm{LCP}})/(1+{\rm{N}}\cdot {\rm{PAR}})$$

PAR is the value of light intensity in the light response curve (or the value of concentration of CO_2_ in CO_2_ response curve). Photo is the net photosynthetic rate. LCP is the light compensation point (or CO_2_ compensation point). E, M and N are parameters. E is also the apparent quantum yield. The dark respiration rate under the light compensation point = E·LCP. The light saturation point is calculated as follows:

$$({\rm{LSP}})=((({\rm{M}}+{\rm{N}})\cdot (1+{\rm{N}}\cdot {\rm{LCP}})/{{\rm{M}})}^{1/2})/-1)/{\rm{N}}.$$

The net photosynthetic rate under the light saturation point (LSP) or CO_2_ saturation point (CSP) can be calculated according to the model.

The data related to the determination of the light curve of chlorophyll fluorescence were automatically fitted according to the model of Eilers and Peeters^5^.

The model of Eilers and Peeters is as follows:

$${\rm{ETR}}\,={\rm{PAR}}/({\rm{a}}\cdot {{\rm{PAR}}}^{2}+{\rm{b}}\cdot {\rm{PAR}}+{\rm{c}})$$

ETR is the electron transport rate of photosynthetic system II. PAR is the fluorescence intensity. The letters a, b and c are parameters.

## Data Availability

Data have been permanently archived: 10.5061/dryad.bg79cnp7h.
